# Spatial and Temporal Variation in Selection of Genes Associated with Pearl Millet Varietal Quantitative Traits *In situ*

**DOI:** 10.3389/fgene.2016.00130

**Published:** 2016-07-26

**Authors:** Cédric Mariac, Issaka S. Ousseini, Abdel-Kader Alio, Hélène Jugdé, Jean-Louis Pham, Gilles Bezançon, Joelle Ronfort, Luc Descroix, Yves Vigouroux

**Affiliations:** ^1^Institut de Recherche Pour le Développement, UMR DIADE, MontpellierFrance; ^2^Institut de Recherche Pour le Développement, NiameyNiger; ^3^University Montpellier II, Place Eugène Bataillon, MontpellierFrance; ^4^University Abdou Moumouni of Niamey, NiameyNiger; ^5^Institut National de Recherche Agronomique, INRA, UMR AGAP, MontpellierFrance; ^6^Institut de Recherche Pour le Développement, IRD, UMR LTHE, GrenobleFrance

**Keywords:** selection, temporal and spatial variability, functional diversity, pearl millet, adaptation to climate variation

## Abstract

Ongoing global climate changes imply new challenges for agriculture. Whether plants and crops can adapt to such rapid changes is still a widely debated question. We previously showed adaptation in the form of earlier flowering in pearl millet at the scale of a whole country over three decades. However, this analysis did not deal with variability of year to year selection. To understand and possibly manage plant and crop adaptation, we need more knowledge of how selection acts *in situ*. Is selection gradual, abrupt, and does it vary in space and over time? In the present study, we tracked the evolution of allele frequency in two genes associated with pearl millet phenotypic variation *in situ*. We sampled 17 populations of cultivated pearl millet over a period of 2 years. We tracked changes in allele frequencies in these populations by genotyping more than seven thousand individuals. We demonstrate that several allele frequencies changes are compatible with selection, by correcting allele frequency changes associated with genetic drift. We found marked variation in allele frequencies from year to year, suggesting a variable selection effect in space and over time. We estimated the strength of selection associated with variations in allele frequency. Our results suggest that the polymorphism maintained at the genes we studied is partially explained by the spatial and temporal variability of selection. In response to environmental changes, traditional pearl millet varieties could rapidly adapt thanks to this available functional variability.

## Introduction

The general test of the theory of evolution led to the conclusion that most polymorphisms are neutral and are transiently polymorphic due to the effect of mutation and drift (Kimura, 1983). However, it is also postulated that some polymorphism may be maintained by variable selection in space and over time (Hedrick, 1986, 2006). The number of documented genes in which polymorphism has been shown to be maintained by this mechanism is limited (Hedrick, 2006). However to date, very few studies have investigated this question *in situ* (O’Hara, 2005; Pemberton, 2010; Mojica et al., 2012; Gratten et al., 2012).

*In situ* studies of selection are rather difficult because variability between years could affect both the strength of selection and the heritability of traits. In sheep, such variability has led to low heritability of birth weight when selection is strong and stronger heritability when selection is weak (Wilson et al., 2006). To assess whether their evolution is simply explained by the effect of drift or whether the effect of selection also plays a role, rather than relying on morphological variation, one can rely directly on the alleles associated with variations in morphological characters.

Studies on pearl millet identified functional polymorphism associated with variations in morphology and as well as in flowering time (Saïdou et al., 2009; Mariac et al., 2011). Some of this polymorphism was associated with the evolution to earlier flowering of traditional varieties over a period of 27 years at a whole country scale (Vigouroux et al., 2011). Pearl millet is an out-breeding crop and traditional varieties sown by farmers from year to year are subjected to selection both imposed by humans and by the environment. The aim of this study was to investigate if selection occurred and varied in the field at the level of varieties. We focused primarily on selection imposed by the environment on varieties and not on that imposed by humans. We consequently did not consider seed selection from one year to the next (Allinne et al., 2008), nor the impact of early thinning, which is known to have a potential selective effect (Couturon et al., 2003). Both of these effects are directly due to human selection. In this paper, we focus on direct environmental effect, *in situ*. For this reason, we sampled seedlings at a later stage along with seeds at maturity to assess changes in overall allele frequency between these two stages.

We analyzed selection over a period of 2 years *in situ* by genotyping more than seven thousand individual plants. We examined the evolution of early and late allele frequency of the two genes in 17 populations over two growing seasons to assess whether or not their evolution was compatible with neutral evolution. As we focused on selection imposed by the environment, we studied the evolution of allele frequency at the seedling stage and at harvest.

## Materials and Methods

### Field Sampling and Plant Material

**FIGURE 1:**
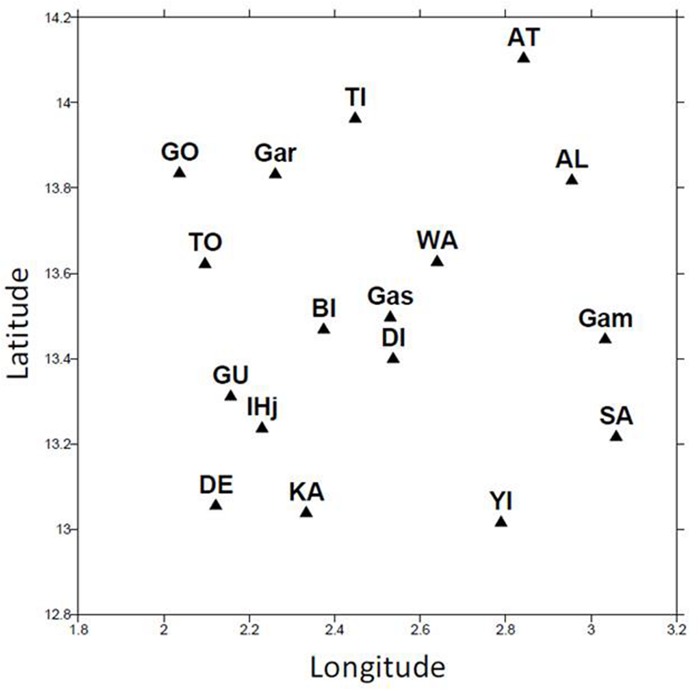
**Location of sampling sites.** A total of 17 different sampling sites were used in the study. These sites were located near Niamey in Niger between longitude 2 and 3.2 and between latitude 13 and 14.2.

Sampling was conducted during two rainy seasons in 2008 and 2009. Seventeen different fields were chosen in an area of 100 km × 100 km around Niamey in Niger (**Figure 1**). The fields were chosen because they were homogenous with respect to soil, their size (mean = 5.7 ha *SE* = 3.4) and the vegetation. No specific permission was required for these experiments, and the experiments did not involve endangered or protected species.

At each site in each year, allele frequencies were estimated at the seedling and seed stage.

One hundred and twenty plants were sampled by choosing the eight closest plants around 15 plots randomly located in a 7,500 m^2^ area chosen in the center of fields.

Seedlings were sampled at the late vegetative stage, in July. Leaf fragments (15 cm^2^) were collected at 4°C and stored at -20°C until DNA extraction. Seeds were sampled just before harvest in October, spikes were randomly picked following exactly the same protocol. Spikes from the entire sample were mixed and threshed to form a bulk sample of seeds from which a subset was grown in the laboratory for DNA extraction. We did not ask the farmer to collect the different spikes, but sampled them ourselves with a trained technician.

The average yield of each farmer’s field was evaluated for each year by counting the number of bundles of spikes harvested. Rainfall data (Supplementary Figure S1) were recorded by pluviographs located close to each field (mean distance from the field = 406 m, *SE* = 551).

### DNA Extraction and Genotyping

DNA was extracted using a modified high-throughput method (Xin et al., 2003; Saïdou et al., 2014). Briefly, 30 mm^2^ of leaves were transferred in a 96-well plate, only very slight crushed with a small plastic stick, and mixed with 50 μl extraction buffer (100 mM NaOH, 2% Tween 20, 10% chelex, pH 10). The plate was then heated at 95°C for 10 min and immediately cooled to 4°C. Then 50 μl of neutralization buffer (100 mM tris, 2 mM EDTA pH 2) was added, mixed and left overnight at 4°C. For PCR amplification, 2 μl of the supernatant was used.

We genotyped either an SNP at the *PgPHYC* gene using a cleaved amplified polymorphic sequence fragment of the gene (see Saïdou et al., 2009 for primer sequences, PCR and digestion conditions and genotype scoring) or a 3 bp indel using a new set of primers for direct PCR amplification and genotyping on an LI-COR sequencer (*PgPHYC*-F5′GCTCTGTTGCGTCACTTG3′; *PgPHYC*-R 5′CTGCTGATCACTCCCAGTAT3′). Two alleles were observed: one at 80 bp and one at 83 bp. According to previous observations, the restriction of size variation polymorphisms is perfectly linked and associated with phenotypic variation (Saïdou et al., 2009, 2014). We also genotyped a 24 bp indel in the *PgMADS11* gene (Mariac et al., 2011) using a simple PCR reaction (*PgMADS11* Forward 5′CCAAAACCAAACCCTAGCAA3′, *PgMADS11* Reverse 5′GTTCAAGAAGGCGGAGGAG 3′). Two alleles were observed, one of 363 bp and one of 387 bp.

The forward primers of *PgPHYC* and *PgMADS11* were synthesized with an M13 forward primer sequence at the 5′-end (5′-ACGACGTTGTAAAACGAC-3′). The PCR of the two genes were performed simultaneously including an M13 labeled primer. The PCR primer concentrations were adjusted to obtain amplification of similar intensity for the two genes (the final concentration of primers in PCR reaction was 0.13 μM of *PgMADS11-*F, 0.36 μM of *PgMADS11-*R, 0.2 μM of *PHYC-* F, 0.2 μM *PHYC*-R, and 0.1 μM of IRDye-700 labeled M13 Primer). The two genes were scored using a LI-COR system using the migration conditions previously described in Allinne et al. (2008). Allele scoring was checked manually by two different people.

A set of nine microsatellite markers was chosen according to their high polymorphism levels and their distribution among the seven linkage groups of pearl millet: PSMP2085, PSMP2202, PSMP2218, PSMP2220, PSMP2237, PSMP2263, PSMP2270, PSMP2271, and PSMP2273 (Mariac et al., 2006). Primers and PCR conditions are described in (Mariac et al., 2006). Microsatellites were genotyped using a LI-COR sequencer (see Allinne et al., 2008 for details). We used Flexibin (Amos et al., 2007) to differentiate alleles.

### Statistical Analysis and F_ST_ Based Test of Selection

For each population, we calculated the genotype and allele frequency of each allele for *PgPHYC* and *PgMADS11*. Differences in allele frequency were assessed using a *G*-test (Sokal and Rohlf, 1981). We calculated the changes in allele frequencies: a difference in a positive value is associated with an increase in the earlier flowering allele, while a negative value is associated with a decrease in the earlier flowering allele.

If selection is acting on a particular functional allele, we expect its allele to show stronger allele frequency changes than a neutral allele. To assess the strength of the change relative to a neutral allele, we need to take into account both the effect of drift and sampling. To do so, we built two F_ST_ distributions describing a neutral expected distribution using a previously described approach (Vigouroux et al., 2011). The first distribution of the F_ST_ value was built based on the F_ST_ value calculated for each allele of the 173 alleles of the nine microsatellite loci. This distribution is hereafter referred to as empirical distribution. We built a second model based on F_ST_ distribution (hereafter referred to as simulated F_ST_ distribution) taking the sampling effect and drift into account. Drift is the direct product of the effective size of the population; consequently, we needed to estimate this parameter. Differences in the allele frequency of microsatellite loci between the seedling sample and the seed sample were used to estimate this effective size using the pseudo-likelihood method (Wang, 2001, 2005). This method makes it possible to derive an expected effective size (Ne) as well as a confidence interval. To be effective, the sampling size needs to be of the same order of magnitude as the unknown effective size. If the sampling size is much lower than the effective size of the population, the method only makes it possible to define the lower bound of the 95% confidence interval, and the estimated effective size or higher bound will be very inaccurate. However, this lower bound leads to over correction for the drift effect and is consequently sufficient to build a conservative distribution.

For the simulation of drift, we used a standard Wright–Fisher model; and considered a single nucleotide polymorphism (SNP) with a frequency *p* drawn from a uniform distribution [0,1]. We simulated variation in allele frequency by simulating the allele frequency after drift associated with an effective size Ne by drawing a binomial law. We then simulated a sample of size n (less than the effective size Ne) from the initial population and the next generation population. From these two samples, we then derived the estimated initial allele frequency, and the estimated final allele frequency. Consequently, this simulation took into account the effect of drift (shaped by Ne) and the effect of sampling (shaped by n). Using the two samples, we were able to easily estimate the differentiation using F_ST_ (Weir, 1996). This simulation was performed using R^1^ and we performed 100,000 simulations. The F_ST_ distribution was then treated as a null distribution against which allele variation on the two genes was tested. We calculated the rank of the differentiation observed for *PgMADS11* or *PgPHYC* in the empirical or the simulation based F_ST_ distribution. This rank, divided by the number of simulations, was used as a *p*-value (Vigouroux et al., 2011). The effective size was estimated for two populations in 2008. For these two populations, the lower bound of the 95% confidence interval for the effective size was used to derive the simulation based F_ST_ distribution. For the other population, we used the smaller lower bound of the two populations to simulate the F_ST_ distribution for the other population. We estimated the effective size of two populations in 2009 and used the same procedure to derive F_ST_ distribution.

Microsatellites have a high mutation rate (Vigouroux et al., 2002), and in this particular case, F_ST_ is not independent of the mutation rate (Vigouroux et al., 2005). But for the generation time considered, the microsatellite mutation rate does not affect F_ST_ estimation (see Vigouroux et al., 2011 for a simulation study).

### Estimation of Selection

The previous analyses proved change in allele frequency beyond drift. We were consequently also able to estimate a selection coefficient using an approximate Bayesian computation approach (Csilléry et al., 2010). In this particular case, we considered that a particular observed allele frequency change is shaped by drift (Ne), sampling (n), and selection (s). We used changes in microsatellite allele frequency to estimate Ne and n is perfectly known, consequently we were able to estimates.

We simulated three genotypes, AA, Aa, and aa, with a frequency q for a. We calculated the genotype frequency associated with a coefficient of selection s and a dominance h: AA: (1-q)^2^/(2hsq(q-1)+1-sq^2^); Aa: 2pq(1-hs)/ (2hsq(q-1)+1-sq^2^); aa q^2^(1-s)/(2hsq(q-1)+1-sq^2^). We simulated the effect of drift based on a multinomial distribution with Ne individuals. We then simulated the effect of sampling with a multinomial distribution with Ns individuals (Ns < Ne). We used a fixed value for Ne, choosing either the average previously reported Ne value or the lower bound of the Ne distribution. ABC approaches are simulation based approaches used to obtain a posterior distribution of a parameter, in this case, s. The prior distribution of s was chosen as a uniform distribution, (0,1). The method was only applied when the F_ST_ approach classified the loci as outliers, i.e., selected. So we only considered loci with s≠0. Based on observed allele frequency q, the genotypes AA, Aa, aa were simulated for a given value of s, Ne, Ns, h. The simulated genotype frequencies were compared to the observed genotype frequency using a khi-square test. The p value of the test was used as the selection criterion. All simulations with a *p*-value of 0.5 or higher were kept. The posterior distribution of the s distribution was based on the simulations that were kept. The median value and the 95% interval were calculated based on this distribution. For validation, the retrieved median value of s was then used to simulate the average frequency of the genotype, and the difference between the simulated frequency and the observed frequency were tested using a *G*-test.

We assessed the ability of the ABC approach to estimate s. We ran a simulation in which we simulated a selection effect (s), a drift effect (Ne), and a sampling size (n). We then estimated a genotype frequency knowing s. We then used the ABC approach to estimate the known value of s. For this simulation, we simulated different values of s: 0.02, 0.05, 0.1, 0.2, 0.3, 0.4, 0.5, 0.7, 0.9. The allele frequency q was set at 0.5. The effective size Ne was set at 100,000 or 300. The sampling size varied from *n* = 100, 200, Ne. The dominance coefficient h was set at 0 or 1. The median value of s estimated using the ABC approach was then compared to the true exact value.

### Field Trial

A field trial was conducted in 2009 using the seeds collected in 2008. The experiment was performed at the International Center of Research for the Semi-Arid Tropics (ICRISAT) station, Sadore, Niger with the agreement of this institution. Two repetitions (plots) were performed at each planting date; and two different planting dates were used: June 15 and July 15, 2009. Each sample in each field trial contained 20 plants, i.e., a total of 340 randomly distributed plants. We recorded flowering time (time from sowing to female flowering) and size of the spike at maturity of each individual plant. An overall measure for each sample (including all 20 individuals) was the weight of the spike, the total seed weight, total dry weight, and 100 seed weight. We performed an analysis of variance using the aov function in R (R Development Core Team, 2008) considering both the effect of samples, date of planting, and replicates. For the analysis of correlation, we used cor.test in R (R Development Core Team, 2008).

## Results

### Changes in Allele Frequency

We retrieved genotypes on 1,659 seedlings for *PgPHYC* and 1,455 seedlings for *PgMADS11* at the 17 sampling sites in 2008. At the seed stage, a total of 1,968 genotypes were obtained for *PgPHYC* and 1,512 for *PgMADS11*. In 2009, 1,904 individual genotypes were obtained for *PgPHYC* and 1,863 for *PgMADS*. Finally, at the seed stage, 1,539 *PgPHYC* genotypes and 1,439 for *PgMADS* phenotype were obtained. A grand total of 7,070 individuals were genotyped for *PgPHYC*, and 6,310 individuals were genotyped for *PgMADS11*. An average 98.4 genotypes were finally obtained for each sampling site, at each stage for each gene.

**FIGURE 2:**
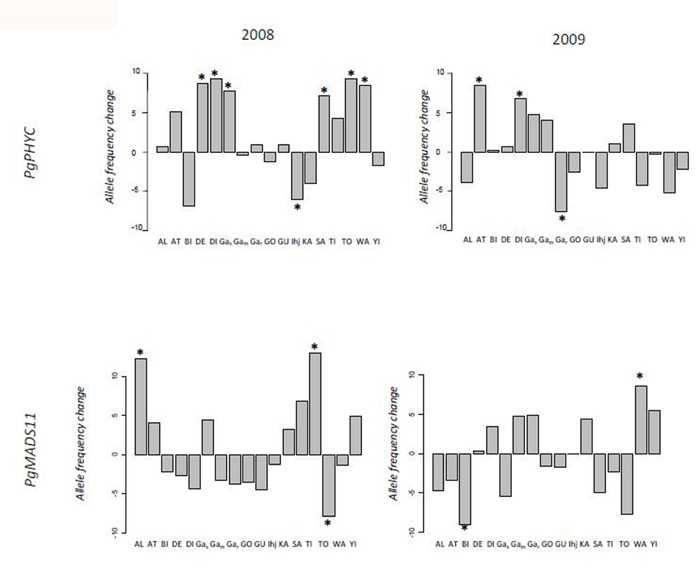
**Differences in allele frequency between the late seedling stage and seed stage of two flowering time genes.** The difference in allele frequency between seedlings and seeds was estimated at 17 sampling sites. Differences in the two flowering time genes *PgPHYC* and *PgMADS11* were assessed during two seasons, 2008 and 2009. A different positive value is associated with an increase in the earlier flowering allele, a negative value with a decrease in the earlier flowering allele. The significance of the difference in frequency was assessed using a *G*-test (*P < 0.05). The 17 different sampling points were Alkama (AL), Ataloga (AT), Billingol (BI), DeberiGati (DE), Diribangou (DI), Gassan Kournie (Gas), Gamonzon (Gam), Gardama Kouara (Gar), Gorougoussa (GO), Guilahle (GU), IHjachere (IHj), Kare (KA), Sandileye (SA), Tiloa Kaina (TI), Tondibia Gorou (TO), Wankama (WA), Yillade (YI).

Allele frequencies between seedlings and seeds (**Figure 2**) differed significantly in the *PgPHYC* gene in seven populations in 2008 (DE +0.088, *p* ≤ 0.015; DI +0.093, *p* ≤ 0.016; Gak +0.078, *p* ≤ 0.029; Ihj -0.061, *p* ≤ 0.036; SA +0.072, *p* ≤ 0.044; TO +0.094, *p* ≤ 0.031; WA +0.085, *p* ≤ 0.031). In 2009, three populations differed significantly in allele frequency in *PgPHYC* (AT +0.085, *p* ≤ 0.014; DI +0.068, *p* ≤ 0.029; Gak -0.075, *p* ≤ 0.044). In *PgMADS*, three populations were significantly different in 2008 (AL +0.123, *p* ≤ 0.007; TI +0.129, *p* ≤ 0.004; TO -0.08, *p* ≤ 0.047,), and two populations were significantly different in 2009 (BI -0.0904, *p* ≤ 0.038; WA +0.086, *p* < 0.046).

The probability of obtaining two or more significant tests out of 17 at a 5% level is 5%, and the probability is 1% with three or more significant tests, and 6.3 10^-7^ with seven or more significant tests. These results suggest that the significant difference observed in 2008 and 2009 in *PgPHYC* and in 2008 in *PgMADS11* is not simply associated with the number of populations investigated.

**FIGURE 3:**
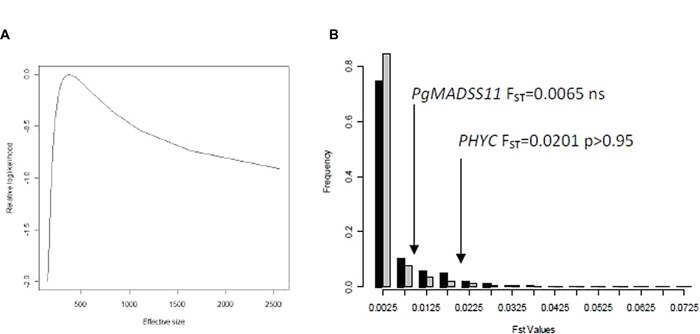
**Estimation of effective size, observed and expected F_ST_ values.** The effective size from seedling to seed was estimated using Wang methods [9] for the Diribangou samples **(A)**. In 2008, nine microsatellites markers were genotyped on 93 seedlings and 144 seeds at this site. The graph shows changes in the relative log likelihood as a function of effective size, the highest likelihood being set to 0. The average estimation was 378, and the lowest 95% interval was 160. The lower interval value was used to simulate a F_ST_ distribution **(B)**. The F_ST_ distribution based on empirical F_ST_ (black) or simulated F_ST_ (gray) are very similar. The significance of the F_ST_ *p*-value was assessed using the simulated F_ST_ distribution for each gene.

However, these different allele frequencies would be expected as the effect of drift if the effective population size is small. We consequently estimated the effective population size (**Figure 3A**; Supplementary Figure S2) using microsatellite datasets for two populations in 2009 and 2008: Diribangou and Tondibia Gorou. In 2008, we estimated the effective size at 296 (95% lower bound: 130) for the Tondibia Gorou population, and 378 (95% lower bound: 160) for the Diribangou population. In 2009, using the same sampling design, we were able to estimate the lower bound of the 95% CI with 216 for Tondibia Gorou and 239 for Diribangou with confidence. The estimated effective size is rather imprecise but was 1,115 for Tondibia Gorou and 8,986 for Diribangou. In 2009, our sample (~100 plants) was much smaller than the lower bound, meaning that, with our present design, the approach based on the change in allele frequency was not powerful enough to assess the average effective size. A bigger sample would be needed to accurately estimate the average effective size. However, these results are sufficiently precise to evaluate the effect of drift on the difference in allele frequency between seedling and seeds. We based our simulation of drift on the lower bound of the confidence interval, leading to conservative tests of significance. The test applied to the population from Tondibia Gorou in 2008 (**Figure 3B**) showed that the observed F_ST_ in *PgPHYC* was extreme in the distribution of the empirical or simulated F_ST_ distribution (*PgPHYC* F_ST_ = 0.0201, *p* < 0.05). No difference was observed in *PgMADS11* (**Figures 2** and **3**). The same results were obtained at the Diribangou site (*PgPHYC* F_ST_ = 0.0201, *p* < 0.05). Using the 95% lower bound interval of the effective size estimated in 2008 and 2009, we built a F_ST_ simulated distribution for the other sites. These analyses confirmed the significant difference in allele frequency at 13 of the 15 sites with a significant *G*-test. The two non-significant F_ST_-tests were observed in 2008 in the IHjachere (IHj) sample for *PgPHYC* and in the Tondibia Gorou sample for *PgMADS11.*

### Estimation of Selection

We developed an ABC approach to estimate the selection coefficient s. This method was effective (Supplementary Figure S3) for a high selection coefficient (*s* > 0.3) for the size of the sample used in this study (Ns~100) and the effective size observed (Ne~300). A bigger sample and higher effective size make it possible to estimate a lower selection coefficient. We then applied this approach at the two original sites (Tondibia Gorou and Diribangou). The selection coefficient estimated for *h* = 0 at the Tondibia Gorou site was 0.49 (95%CI 0.06–0.75). The selection coefficient estimated at the Diribangou site was 0.44 (95%CI 0.06–0.69). These estimated coefficients were then used to calculate the expected frequency of the genotype and to compare these frequencies with the observed class of genotypes. The selection coefficients were in agreement with the frequency observed at Tondibia Gorou (*χ*^2^ = 0.30, dof = 2, *p* = 0.85) and at Diribangou (*χ*^2^ = 1.69, dof = 2, *p* = 0.43).

### Morphological Analyses

Field trail analyses revealed a significant difference (**Table 1**) between varieties in flowering time (June 15, *F*_16,600_ = 13.9, *p* < 0.001; July 15 *F*_16,595_ = 9.3, *p* < 0.001) and in spike length (June 15, *F*_16,601_ = 7.3, *p* < 0.001; July 15 *F*_16,595_ = 9.0, *p* < 0.001). As only aggregated data were available for yield, biomass and 100-seed weight, we calculated the correlation between allele frequency and each of these morphological characters as well as for flowering time and spike length. Significant correlations were found for yield with *PgPHYC* allele frequency at both sowing dates (June 15, *R* = -0.69, *p* < 0.006; July 15, *R* = -0.69, *p* < 0.02), but only for the earlier seedling for *PgMADS11* (June 15, *R* = -0.60, *p* < 0.012; July 15, *R* = -0.34, *p* = 0.19). The correlation with biomass tended to be only significant for *PgPHYC* and at the early planting date (June 15, *R* = -0.51, *p* < 0.05). A significant correlation for *PgMADSS11* and flowering time was only found at the late planting date (*R* = -0.48, *p* < 0.05). The other correlations were not significant.

**Table 1:** Correlation between allele frequency and phenotypic variations.

Phenotype	*PgPHYC* allele frequency		*PgMads11* allele Frequency	
Planting date	June 15	July 15	June 15	July 15
Flowering time	*R* = -0.06 *p* = 0.85	*R* = -0.44 *p* = 0.08	*R* = -0.38 *p* = 0.14	***R* =** -**0.48 *p* < 0.05**
Spike length	*R* = -0.18 *p* = 0.48	*R* = -0.32 *p* = 0.21	*R* = -0.28 *p* = 0.27	*R* = -0.36 *p* = 0.15
Yield	***R* =** -**0.69 *p* < 0.006**	***R* =** -**0.69 *p* < 0.002**	***R* =** -**0.60 *p* < 0.012**	*R* = -0.34 *p* = 0.19
Biomass	***R* =** -**0.51 *p* < 0.05**	*R* = -0.33 *p* = 0.19	*R* = -0.48 *p* = 0.053	*R* = -0.44 *p* = 0.08
Hundred seed weight	*R* = -0.27 *p* = 0.29	*R* = -0.20 *p* = 0.44	*R* = -0.05 *p* = 0.86	*R* = -0.13 *p* = 0.61

*Frequencies of the two alleles observed at the two genes were correlated with flowering time, spike length, yield, total biomass, and 100 seed weight. Bolded values are significant at *p* < 0.05.*

**FIGURE 4:**
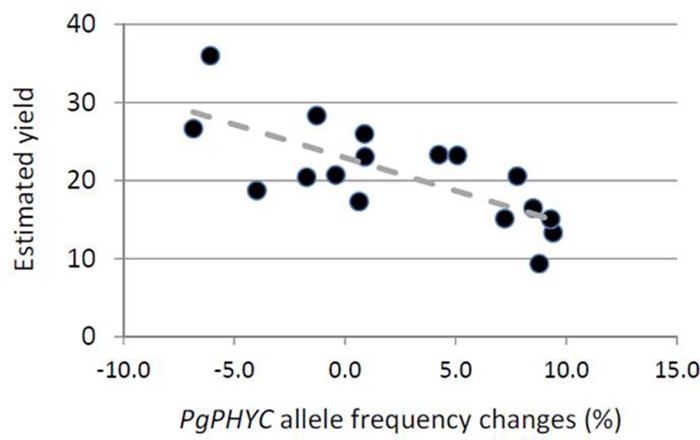
**Relationship between changes in *PgPHYC* allele frequency and estimated yield.** For each sampling site, we plotted the change in the *PgPHYC* allele frequency and estimated yield. Yield was estimated *in situ* based on the number of bundles of spikes per hectare. The relationship is significant (*R* = -0.76, *p* < 0.001).

Finally, in 2008 and 2009, we recorded the yield of all the populations in the field. We used this estimation of *in situ* yield to check whether or not the variation in allele frequency was linked with yield. We only found (**Figure 4**) a significant negative correlation for *PgPHYC* in 2008 (*R* = -0.76, *p* < 0.001). No significant correlation was found for 2009 or *PgMADS11*.

## Discussion

The two genes *PgMADS11* and *PgPHYC* have previously been shown to have an effect on several traits including flowering time and spike length (Saïdou et al., 2009; Mariac et al., 2011; Vigouroux et al., 2011). A recent study found also an association between *PgPHYC* and panicle harvest index (Sehgal et al., 2015). For both genes, we detected a significant effect on yield in the field trials. However, one of the effects was not significant for *PgMADS11* at the later planting date (July 15). These correlations (here and in Sehgal et al., 2015) suggest that variations in these genes may have a direct impact on fitness (yield in an agricultural setting). However, this effect could be influenced by other parameters, for example, planting date. In this particular case, depending on the emergence of the plant, polymorphism could be associated with fitness or not.

In our study, effective size strongly varied between years. This result is not surprising, since in the Sahel, crop failure is frequently observed and climate variability is very high. Total crop failure will actually lead to an effective size of zero, and the maximum value one could expect is the total number of individuals randomly breeding in the field. Taking the effect of drift into account, our study suggests a variation in the selection of genetic markers associated with quantitative traits. Our results suggest that this variation varied from positive selection in 1 year to neutral or negative selection in the other. If variation in allele frequency is associated with selection, then one might expect a correlation between what will measure the strength of selection (difference in allele frequency between seedlings and seeds) and the overall fitness of the population. We actually found a significant correlation between variation in *PgPHYC* allele frequency and yield in the field in 2008 (**Figure 4**). Such a correlation would be detected if and only if a large number of sampling sites had significant changes in allele frequency, i.e., is associated with selective events. We actually only observed such a situation for *PgPHYC* in 2008. In the other year for *PgMADS11*, few sampling sites showed significant changes in allele frequency. It should be noted that majority of the field studies/gene studies over 2 years (53/68) showed no significant change in *PgPHYC* and *PgMADS11* allele frequencies. And using the F_ST_ based test of selection, 81% (55/68) of the field studies/gene pairs were considered to have changes in allele frequencies that did not differ from a neutral pattern.

In our study, the point estimated selection coefficient is high (~0.44–0.49). However, since the confidence interval is relatively high, our results are compatible with a lower selection coefficient. A high coefficient of selection was also observed *in situ* in a field study on mice (Linnen et al., 2013). Such a high selection coefficient could rapidly lead to the fixation of an allele in a given population. However, several factors certainly helped maintain polymorphism in the present study: the variability of selection (in space and over time), the effect of gene flow (Allinne et al., 2008), and, in our context, human counter selection. One of the first variability factors to be considered is the variability of the environmental conditions. The Sahelian climate is known to vary considerably from year to year, as actually observed in our field study (Supplementary Figure S1). Moreover, as previously discussed, there is a complex interaction between allele frequency, environment and fitness. Again this interaction could play a significant role in maintaining polymorphism: polymorphisms with such interaction do not always appear to be associated with fitness, and in our study mostly appeared to be “neutral”. Another factor that certainly plays a role is human selection. We need to underline that our study was performed during a short period of time during which humans cannot not have a direct effect on changes in allele frequency, i.e., between the late seedling stage and seed maturity. In a previous study, we demonstrated that *PgPHYC* is associated with slightly shorter spikes (Saïdou et al., 2009, 2014). Human selection after harvest might favor longer spikes, which are associated with higher yields, and with what farmers consider to be the normal morphology of their varieties. It that case, human interaction might also counter environmentally imposed selection. Moreover, when crop failure is high, farmers import new seeds sometimes from a distance (Allinne et al., 2008), which might also provide an opportunity for environmental selection. To decipher the role of these different types of selection, future experiments should include selection imposed by the environment and that imposed by humans. Here we only focused on selection imposed by the environment.

In this study, one factor that was possibly not controlled was pollen gene flow from neighboring fields. Few pollen gene flow studies have been conducted in pearl millet but data are available on maize, a wind pollinated crop that closely resembles pearl millet. Field studies on maize suggest that gene flow is as low as 1% at a distance of 60 m (Bateman, 1947). Based on this figure, if we considered the biggest difference between populations for *PgPHYC* frequency (24%), a gene flow of 1% would lead to a 0.24% change in allele frequency. It is thus unlikely that pollen gene flow between neighboring fields would change *PgPHYC* or *PgMADS11* allele frequency to the level observed in this study.

The study of selection in an ecological context requires the fulfillment of several conditions. Selection can only be assessed if it is high enough to outperform the effect of drift. So a large population is needed. The size of the sample needs to be adjusted so that the effect of selection can be identified. In this study, we used a sample of around 100 plants, i.e., 200 chromosomes. A bigger sample would be needed to detect small changes in allele frequency and a lower selection coefficient. It also certainly means that the detection of selection in field conditions will be particularly difficult unless either the selection coefficient is very high and/or the population size (and hence the sample) is sufficiently large. This requirement may explain why observations of selection maintained polymorphism are so rare.

## Conclusion

The results of study suggest variability of selection on alleles associated with phenotypic variation *in situ*. Such selection could certainly lead to the ultimate fixation of an allele but the variability of the selection in space and over time could certainly maintain such functional variation. Selection for these genes could be rapid in response to environmental changes. In addition, we only considered environmentally imposed selection and in the present case, human selection may also have had a major impact on the allele frequency of the two genes. A better understanding of the dynamic constraints of such a system needs to incorporate environmental as well as human selection pressures.

## Author Contributions

CM, J-LP, LD, IO, and YV designed the research. CM, IO, HJ, KX, JR, GB performed the research, contributed reagent and feedback, CM and YV performed the statistical analysis, IO and YV developed the ABC model for s estimation, CM, IO, and YV wrote the paper.

## Conflict of Interest Statement

The authors declare that the research was conducted in the absence of any commercial or financial relationships that could be construed as a potential conflict of interest.

## Footnotes

1 https://www.r-project.org/
