# Supplementary material for: Spatial and Temporal Variation in Selection of Genes Associated with Pearl Millet Varietal Quantitative Traits In situ
Source: Front Genet. 2016 Jul 26;7:130. doi: 10.3389/fgene.2016.00130 (PMC4960089; doi:10.3389/fgene.2016.00130)
Supplement: Supplementary file 1 [file Image_2.PDf]

Supplementary data

Figure S1. Spatial variability of annual rainfall in the sampled area in 2008 and 2009.

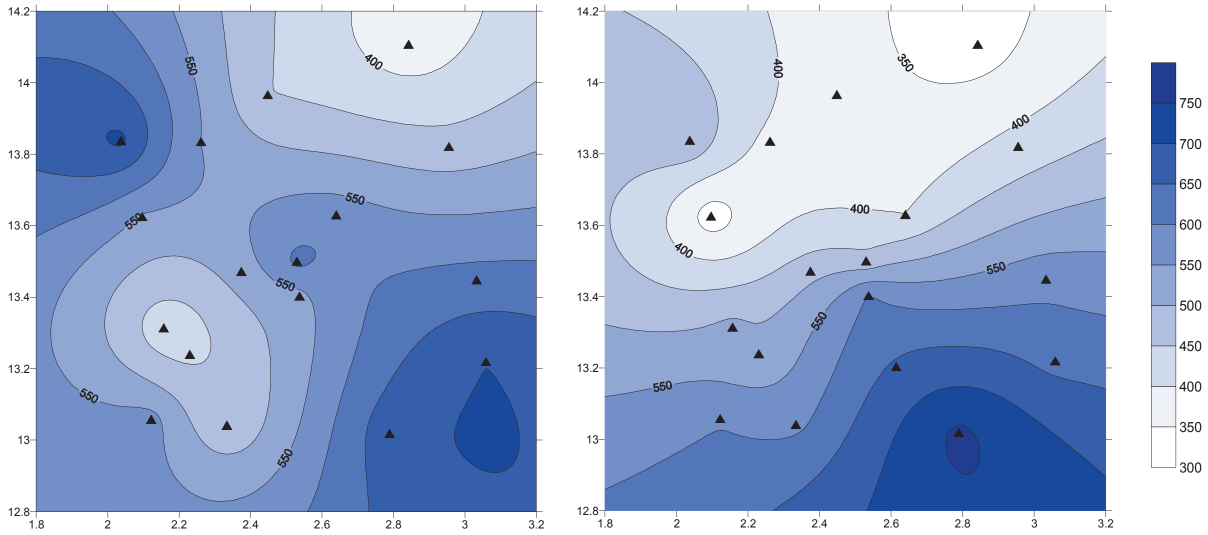

The average isohyets for each year 2008 and 2009 were calculated for the sampling area. Strong variation in rainfall is observed both spatially and from year to year.

Figure S2. Estimation of effective size.

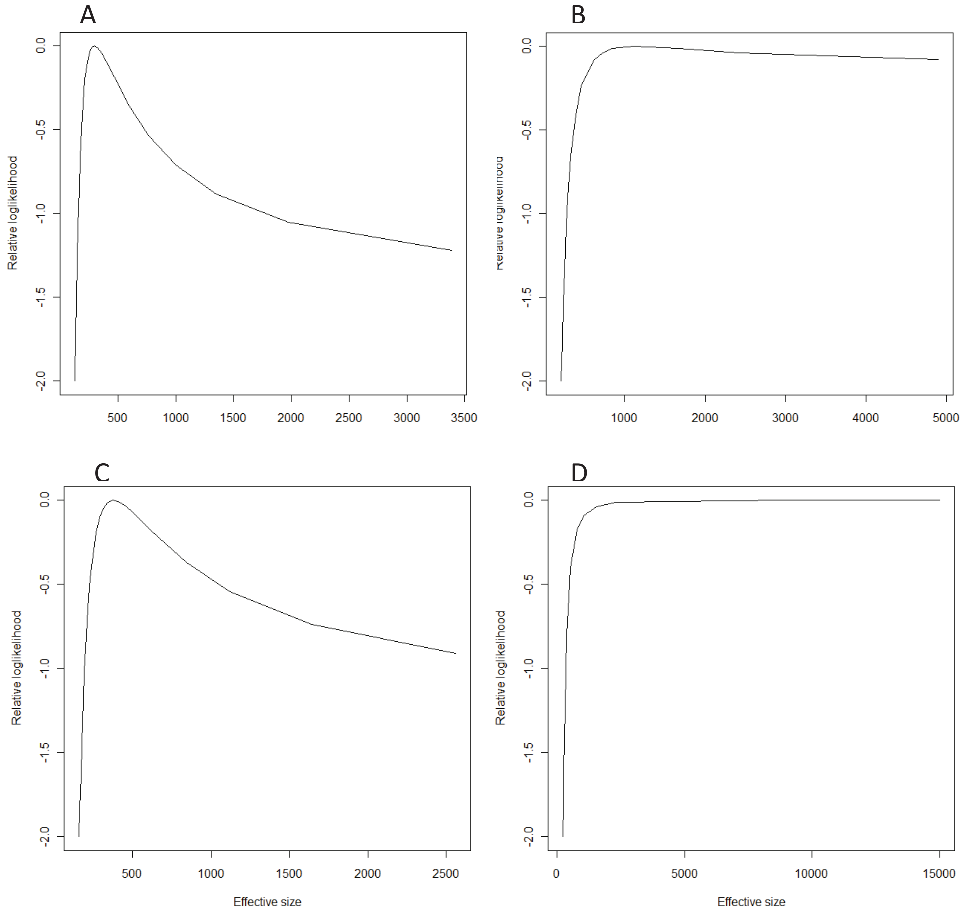

The effective size was assessed using microsatellite locus for two sites Tondibia Gorou (A,B) and Diribangou (C,D) for 2008 (A,C) and 2009 (B,D). The effective size is poorly estimated in 2009 because the number of plant sampled (around 100) is much lower than the effective size. However, for 2009, we could calculate the lower bound of the distribution and used it to assess whether evolution of allele frequency is simply reflected by the effect of drift.

Figure S3. Comparison of s simulated and s estimated using an ABC approach.

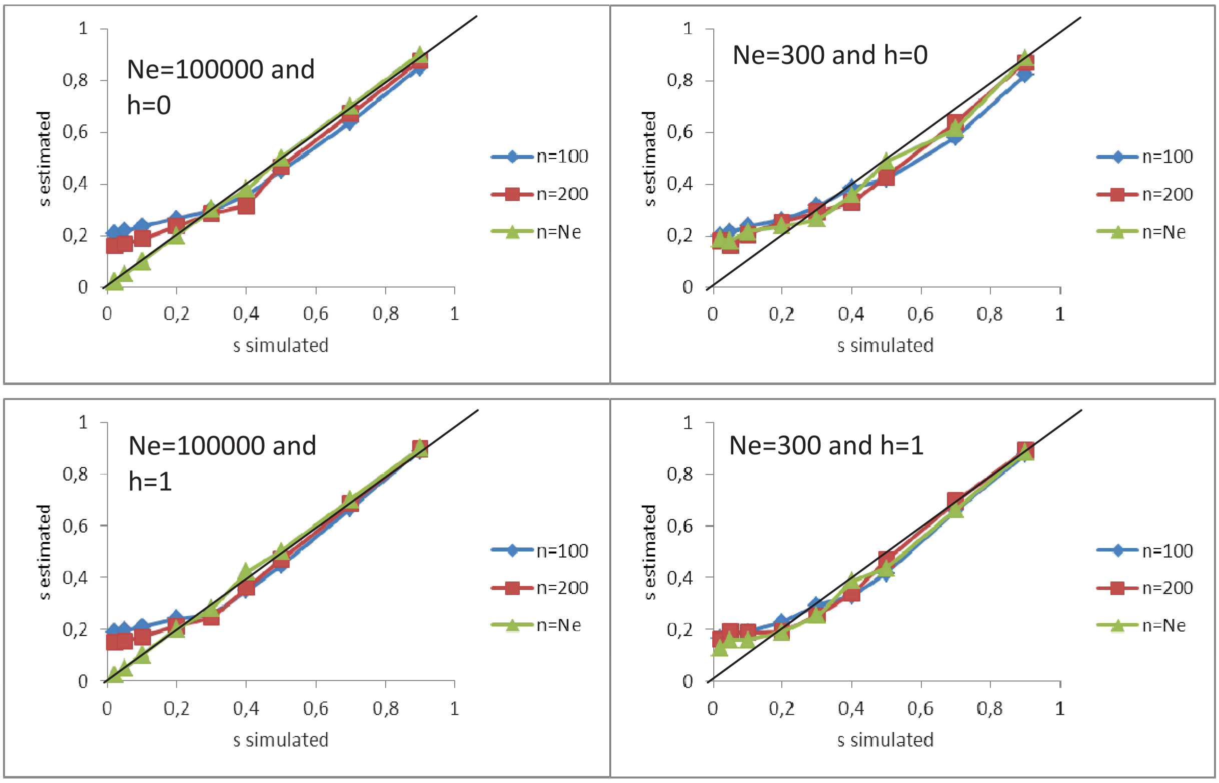

Nine different values of s were used to simulate a dataset (s equal to 0.02; 0.05; 0.1; 0.2; 0.3; 0.4; 0.5; 0.7 and 0.9). The median value of s estimated using the ABC approach was then calculated for different sampling sizes Ns (Ns=100; Ns=200 and Ns=Ne=100000) and two values of the effective size (Ne=100000, Ne=300). The analysis was performed with two dominance coefficients h (h=0 or h=1). The value of s estimated is plotted against the value of s simulated. The x=y line in black represents the expected value of the s estimated knowing s evaluated if the estimation is perfect. The methods are generally effective for s>0.2 or 0.3 whatever the sampling size, the effective size and the dominance coefficient.

Supplementary Material

Spatial and temporal variation in selection of genes associated with pearl millet varietal quantitative traits *in situ*

Cédric Mariac, Issaka Salia Ousseini, Abdel-Kader Alio, Hélène Jugdé, Jean-Louis Pham, Gilles Bezançon, Joelle Ronfort, Luc Descroix, Yves Vigouroux

*** Correspondence:** Corresponding Author: yves.vigouroux@ird.fr.
